# Low-cost alternative biodiesel production apparatus based on household food blender for continuous biodiesel production for small communities

**DOI:** 10.1038/s41598-021-93225-5

**Published:** 2021-07-05

**Authors:** Wijittra Wongjaikham, Doonyapong Wongsawaeng, Vareeporn Ratnitsai, Manita Kamjam, Kanokwan Ngaosuwan, Worapon Kiatkittipong, Peter Hosemann, Suttichai Assabumrungrat

**Affiliations:** 1 Research Unit On Plasma Technology for High-Performance Materials Development, Department of Nuclear Engineering, Faculty of Engineering, Chulalongkorn University, 254 Phayathai Road, Pathumwan, Bangkok, 10330 Thailand; 2 Department of Science and Mathematics, Faculty of Science and Technology, Rajamangala University of Technology Tawan-Ok, Chonburi, 20110 Thailand; 3 Division of Chemical Engineering, Faculty of Engineering, Rajamangala University of Technology Krungthep, Bangkok, 10120 Thailand; 4 Department of Chemical Engineering, Faculty of Engineering and Industrial Technology, Silpakorn University, Nakhon Pathom, 73000 Thailand; 5 Department of Nuclear Engineering, Faculty of Engineering, University of California at Berkeley, Berkeley, 94720 USA; 6 Center of Excellence in Catalysis and Catalytic Reaction Engineering, Department of Chemical Engineering, Faculty of Engineering, Chulalongkorn University, Bangkok, 10330 Thailand; 7 Bio-Circular-Green-Economy Technology & Engineering Center, Faculty of Engineering, BCGeTEC, Chulalongkorn University, Bangkok, 10330 Thailand

**Keywords:** Biodiesel, Chemical engineering

## Abstract

Fatty acid methyl esters (FAMEs) are sustainable biofuel that can alleviate high oil costs and environmental impacts of petroleum-based fuel. A modified 1200 W high-efficiency food blender was employed for continuous transesterification of various refined vegetable oils and waste cooking oil (WCO) using sodium hydroxide as a homogeneous catalyst. The following factors have been investigated on their effects on FAME yield: baffles, reaction volume, total reactant flow rate, methanol-oil molar ratio, catalyst concentration and reaction temperature. Results indicated that the optimal conditions were: 2000 mL reaction volume, 50 mL/min total flow rate, 1% and 1.25% catalyst concentration for refined palm oil and WCO, respectively, 6:1 methanol-to-oil molar ratio and 62–63 °C, obtaining yield efficiency over 96.5% FAME yield of 21.14 × 10^–4^ g/J (for palm oil) and 19.39 × 10^–4^ g/J (for WCO). All the properties of produced FAMEs meet the EN 14214 and ASTM D6751 standards. The modified household food blender could be a practical and low-cost alternative biodiesel production apparatus for continuous biodiesel production for small communities in remote areas.

## Introduction

Fossil fuels and their derivatives are the main sources for transportation, electricity generation and agriculture. However, it is widely accepted that fossil fuel leads to large greenhouse gas emissions^1^ and particulate matter causing environmental consequences. Alternative non-petroleum-based fuels such as biodiesel have received considerable attention recently due to the fact it is renewable, which is more environmentally-friendly than petroleum-derived diesel fuels in terms of reducing exhaust emissions and high biodegradability^2^. Biodiesel is a compound of mono-alkyl esters derived from various types of vegetable oils, lard, waste cooking oil (WCO), and unicellular or multicellular plants including certain species of algae^3^. In the first generation of biofuels, the raw material is extracted from crop plants, for instance, palm, soybean, corn, sunflower, rapeseed and so on^4,5^. Different regions use different oils as a feedstock for the production of biodiesel. Palm oil is usually used in tropical countries (Southeast Asia) while soybean and rapeseed oil are widely used in the USA and European countries, respectively^6^. In 2018, Thailand was ranked as having the third largest palm oil plantation in the world with a planting area of over 8.5 × 10^3^ km^2^ and palm oil production of up to 15 million tons^7^. Nevertheless, domestic palm oil price tends to decrease. Therefore, the use of palm oil as the feedstock to produce biodiesel will stimulate the economy, help farmers indirectly and increase the value-added of palm oil. Furthermore, the molecular structure of biodiesel derived from vegetable cooking oil is redefined leading to the reduction of greenhouse gas emissions^8^ which is one of the principles of sustainable biofuels.

Most commercial-level biodiesel production processes commonly use vegetable oil reacting with alcohol, mostly methanol, through a chemical process called transesterification to produce fatty acid methyl esters (FAMEs) and glycerol together with conventional homogeneous base or acid catalysts^9,10^. For the base catalysts, potassium hydroxide and sodium hydroxide have been preferred by the industry because of their low cost, high reaction rates and faster process^10,11^. Generally, biodiesel synthesis using alkali catalysts requires 1–4 h under different reaction temperatures^12^. Homogeneous acid catalysts that have been used are such as sulfuric acid, sulfonic acid, hydrochloric acid and phosphoric acid. Acid-catalyzed biodiesel production accompanies the reaction through esterification which does not lead to soap formation, but the reaction is quite slow, requires high temperature and high alcohol to oil molar ratio, and may cause reactor corrosion^10^. Miao et al.^13^ indicated that 2.0 M trifluoroacetic acid reacting with methanol to soybean oil molar ratio of 20:1 at 120 °C after 5 h of operation gave the FAME yield of 98.4%. On the other hand, heterogeneous catalysts have been applied for biodiesel synthesis since they offer repeated usability and are easily separated from the end products. The drawbacks of them come not only from excessive conditions of elevated temperature and pressure but also from product contamination from the leaching process^14^. The use of an enzymatic catalyst has become one of the alternative methods because of being more eco-friendly than chemical catalysts; however, the production cost is still much higher than traditional catalysts and the reaction should be performed at low temperatures with a long reaction time^1^. With these regards, the use of an alkali catalyst in the present work offers a simple operation and can shorten the reaction time for biodiesel production.

Typically, when mixing oil with methanol, two-liquid phases will be generated, so advanced intensification technologies have been designed and developed to improve the miscibility of the liquids and to enhance speed, heat and mass transfer, reaction time, energy efficiency and cost effectiveness over the mechanical stirring method. Examples of using intensification technologies for continuous biodiesel production are as follows. Choedkiatsakul et al.^15^ used the microwave system to continuously produce biodiesel from palm oil. They found that operation at a methanol to oil molar ratio of 12, a microwave heating power of 400 W, reaction temperature of 70 °C, and NaOH catalyst loading of 1 wt% of oil gave an ester content of 99.4% in 1.75 min residence time. Mostafaei and the group^16^ optimized the continuous production of biodiesel with response surface methodology by using an ultrasonic reactor. The biodiesel yield of 91.6% was obtained from 50 mL/min flow rate and power consumption of 102.8 W. In addition, a microchannel reactor^17^ showed fast chemical reactions because heat and mass transfer was enhanced through improved surface area to volume ratio. Other intensified reactors to produce biodiesel have been employed, for instance, hydrodynamic cavitation^18,19^, oscillatory^20^, static mixer^21^, spinning disc reactor^22^ and reactive distillation reactor^23^. According to selected pieces of research above, all of them show some gaps. For example, most technologies require expensive equipment pieces that lead to costly maintenance expenses, consume large energy and limit the scale-up production, as well as being infeasible for small communities, while some conditions require high pressure and high temperature causing safety concerns.

Moreover, a conventional process of biodiesel production in communities relies on a batch process. The batch production has some drawbacks such as the requirement of a larger reaction volume and lower efficiency due to the natural start-up and shutdown system^24^. To eliminate these limitations, continuous biodiesel production processes have been put forward to increase the production efficiency over time and large-scale production aspects. Besides, a continuous process offers several benefits compared to batch production. It requires a smaller investment to obtain the same quantity of biodiesel and produces more biodiesel per unit of labor. Therefore, the present research aimed to use a high-power kitchen food blender modified with a stainless steel chamber to produce biodiesel on a continuous basis for small communities based on vegetable oils and WCO. Effects of baffle, reaction volume and flow rate, reactant molar ratio, catalyst loading, reaction temperature and reactant discharge position on FAME yield were investigated. FAME properties and yield efficiency were also measured. This system offers the compact size of the reactor, simplicity of set up, control, operation and maintenance for biodiesel production.

## Experimental

### Materials

Various fresh refined vegetable oils and waste cooking oil (WCO) were used as the feedstocks for continuous biodiesel production. Relevant properties of the vegetable oils and WCO are presented in Supplementary Table S1. The feedstocks: refined palm oil, soybean oil, corn oil and canola oil of Sime Darby Oils Morakot Co., Ltd., Thailand, and refined sunflower oil of Thanakorn Vegetable Oil Products Co., Ltd., Thailand, were purchased from a local supermarket in Bangkok, Thailand. The WCO was provided by a canteen in the Faculty of Engineering, Chulalongkorn University, Bangkok, Thailand. All chemicals were of analytical reagent grade and were used as received. 99.8% methanol and 97% sodium hydroxide were supplied from Kemaus. 99.5% n-heptane and > 99.0% methyl heptadecanoate (analytical standard) were obtained from Ajax FineChem and Sigma-Aldrich, respectively.

### Equipment details and set up

The high-power food blender of 1200 W, 220 V, 50 Hz, 25,000 rpm maximum (Otto brand, model: BE127A) was upgraded with a 304 stainless steel chemical reactor. The supplied plastic blending bowl that came with the blender was replaced with the stainless steel rector to ensure long-term durability and safety. The cylindrical-shaped reactor was 19.8 cm in height, 15 cm in diameter and 0.3 cm in thickness. The maximum capacity of the reactor is 3500 mL. It was insulated with cotton sheets to reduce heat loss to the environment and was installed with 4 baffles of 1.3 cm in width. No modification to the stainless steel impeller shown in Supplementary Fig. S1 was made; it was the original one supplied with the blender. One side of the reactor had several ports installed with thermocouples and the opposite side was equipped with several discharge ports corresponding to the desired mixture volumes inside the reactor (1000–3000 mL). The lid of the reactor had an inlet port for reactant injection, a quartz viewport to allow visual observation inside the reactor, and a condenser port to condense vaporized methanol back into the reactor as well as to ensure atmospheric-pressure operation inside the reactor at all time. For the reactant injection port, a small stainless steel tube was connected to it to discharge the reactants at approximately 1.5 cm above the center of the impeller. A salient design details of the equipment are listed in Supplementary Table S2 and a diagram of the setup for continuous transesterification is shown in Supplementary Fig. S2.

### Continuous FAME production from refined palm oil

The effect of reaction volume (1000, 1500, 2000, 2500 and 3000 mL) was first studied, followed by the effect of reactant total flow rate (25, 50 and 75 mL/min) on FAME yield with the conditions of NaOH loading of 1 wt%, methanol to oil molar ratio of 6:1 and reaction temperature of 62–63 °C. After obtaining the optimal parameters of reaction volume and total feed flow rate, the effects of methanol to oil molar ratio (3:1, 6:1 and 9:1), concentration of NaOH (0.75, 1 and 1.25 wt%) and reaction temperature (50–65 °C) were evaluated. Also, the effect of reactant discharge position on FAME yield was explored by adjusting the position from the center of the reactor to the wall of the reactor. A control transesterification without the catalyst was also performed.

In all runs, the oil and the catalyst dissolved in methanol were simultaneously loaded into the reactor and the solution was heated to 62–63 °C for 15 min by energizing the food blender. Methanol with the dissolved catalyst and oil were then fed into the reactor, with each flow rate separately controlled by a peristaltic pump, and the mixture started to flow out through one of the discharge ports left open at the desired reaction volume. A minor variac adjustment was performed periodically to maintain the reaction temperature of 62–63 °C. Every studied condition, except for the negative control, was performed in duplicate. The product was sampled at 15, 30, 60 and 90 min. Sampled products were centrifuged for 5–7 min to physically separate the FAME layer for further yield analysis. Finally, the products were purified by washing impurities with DI water and heated at a temperature of 110 °C to remove the remaining water.

To determine the effect of insulation on FAME production, refined palm oil and WCO were used as feedstocks under the optimal conditions with a total reactant flow rate of 50 mL/min. The cotton sheets were removed from the chemical reactor and a fan was used to help dissipate heat from the system. A schematic of the overall biodiesel production process was presented in Supplementary Fig. S3.

### Continuous FAME production from various types of edible vegetable oils

The effect of various refined vegetable oil types on oil conversion to FAME was evaluated using the best set of process parameters obtained from FAME production from refined palm oil.

### Continuous FAME production from WCO

When using WCO as a feedstock for transesterification, water content and free fatty acid (FFA) content were important parameters. Under base-catalyzed transesterification, the presence of water leads to hydrolysis reaction of the base catalyst caused by soap formation with triglycerides, free fatty acid, and FAME resulting in reduced FAME yield^25^. Moreover, a neutralized reaction of a base catalyst and FFA would lead to losses of a catalyst^26^. WCO used in biodiesel production should not have the FFA content greater than 2%^27^, or the WCO must be pre-treated with the esterification process before being used as a feedstock. To determine the FFA content of the WCO used in the present study, the traditional acid–base titration analysis was employed. The sampled WCO in a hot 2-propanol solution was titrated by potassium hydroxide using phenolphthalein as an indicator^28^. The FFA content in the WCO was measured to be 1.21% as shown in Supplementary Table S1 as well as density, acid value and kinematic viscosity, so it can be used without an esterification pretreatment. The WCO still needed to be filtered by a fine-mesh sieve to extract food and other residues. The dewatering process was then performed by heating the oil at 110 °C for 30 min. Subsequently, the WCO was passed through a 500 µm filter for final purification. The water concentration of WCO was determined by Karl Fischer coulometric titration method presented below 0.05 wt% following ASTM D6751 standard^29^.

Continuous FAME production based on WCO was studied using the optimal conditions of refined palm oil. Because the alkali catalyst was used to neutralize the FFA present in WCO, the catalyst concentrations of 1.0, 1.25 and 1.5 wt% of oil were also studied.

### FAME identification

FAME yield was determined following the EN 14103 standard^30^, using Shimadzu GC 2010 Plus. This GC system was installed with a flame ionization detector and a DB wax capillary column. FAME yield was evaluated according to Eq. (1):

1 $${\text{FAME}}\;~{\text{yield~}}\left( \% \right) = ~\frac{{\left( {\sum {\text{A}}} \right)~ - {\text{A}}_{{{\text{IS}}}} }}{{{\text{A}}_{{{\text{IS}}}} }}~ \times ~\frac{{{\text{C}}_{{{\text{IS~}}}} \times ~{\text{V}}_{{{\text{IS}}}} }}{{\text{m}}}~ \times ~100~\%$$

where $\sum {\text{A}}$ = total peak area, ${\text{A}}_{{{\text{IS}}}}$ = peak area of methyl heptadecanoate (internal standard), ${\text{C}}_{{{\text{IS}}}}$ = concentration of methyl heptadecanoate (mg/mL), ${\text{~V}}_{{{\text{IS}}}}$ = volume of methyl heptadecanoate (mL), and ${\text{m}}$ = mass of FAME sample (mg).

### Kinetic model

Normally, vegetable oils comprise 90–98 wt% of triglyceride (TG) and a small quantity of diglyceride (DG) and monoglyceride (MG)^31^. Reversible transesterification of vegetable oil and mono-alcohol to produce alky esters and glycerol. Many researchers assume that excess alcohol can shift the reaction forward and that the loss of alcohol is minimized while its concentration remains unchanged. So, the reverse reaction and the intermediate products (e.g., DG and MG) can be ignored^32,33^. In this study, the alkaline-catalyzed transesterification of refined palm oil and WCO was tested through the first order kinetic model. The 1 wt% NaOH (for refined palm oil) and 1.25 wt% (for WCO) loading at 50–65 °C were the conditions to determine the model.

The first-order kinetic model can be simplified from the pseudo-first-order model as Eq. (2)^22^

2 $$\frac{{{\text{X}}_{{\text{A}}} }}{{\left( {1 - {\text{X}}_{{\text{A}}} } \right)}} = {\text{k}}\tau$$

where ${\text{x}}_{{\text{A}}}$ is the palm oil conversion (FAME yield), ${\text{k}}$ is the rate constant, and $\tau$ is the residence time.

### Arrhenius parameters

The activation energy for transesterification can be determined through the Arrhenius equation, which establishes a relationship among the reaction rate constant (*k*), reaction temperature (*T*) and the activation energy (*E*_*a*_). By plotting ${\text{ln}}\left( k \right)$ vs. $1/T$, the slope and the Y-interception give $- \frac{{E_{a} }}{R}$ and ${\text{ln}}\left( {k_{0} } \right)$ (the frequency factor), respectively.

### FAME properties

The physicochemical properties such as density, kinematic viscosity, acid value and cloud point of the produced FAMEs under optimal conditions of refined palm oil and WCO were determined per the EN 14214 and ASTM D 6751 standards.

### FAME yield efficiency

Yield efficiency or energy efficiency was determined to allow power efficiency comparison of different reactor types. It is simply defined as the rate of FAME production divided by the power supply as expressed in Eq. (3)^22^:

3 $${\text{Yield~}}\;{\text{efficiency}}\;~\left( {\frac{{\text{g}}}{{\text{J}}}} \right) = \frac{{{\text{Rate~}}\;{\text{of}}\;{\text{FAME}}\;{\text{production~}}\;\left( {\frac{{\text{g}}}{{\text{s}}}} \right)}}{{{\text{Power~}}\;{\text{supply}}\;~\left( {\frac{{\text{J}}}{{\text{s}}}} \right)}}~$$

## Results and discussion

### FAME production based on refined palm oil

#### Effect of baffles in reactor

**Figure 1:**
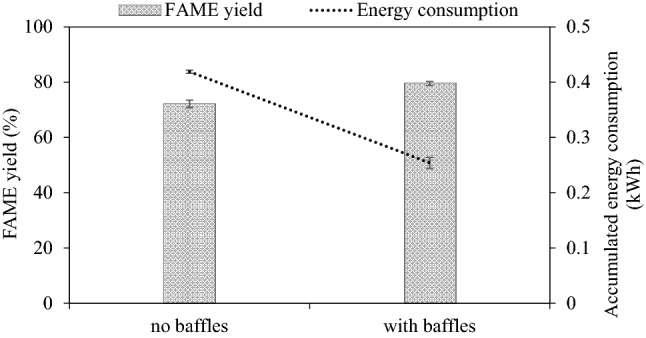
Effect of baffle plates on steady-state FAME yield (refined palm oil, 1000 mL, 25 mL/min, 1 wt% NaOH, 6:1 MeOH: oil molar ratio, 62–63 °C).

For large rectors, the installation of baffles is common practice to provide more effective mixing and heat transfer in the reactor^34^. Without baffles, the fluid would spin freely without achieving good mixing and reaction yield. To ensure that the designed chemical reactor with the rapidly-spinning impeller does require baffles similar to conventional chemical reactors with slower spinning speeds, the effect of baffles was studied here. The reactor was studied with and without appropriate longitudinal flow baffles on the lid extending into the reactor. The dimensions of the baffles were as follows^35,36^: number of baffles = 4, width = 12.5 mm, length = along the reactor terminating at 10 mm from the bottom, off-wall distance = 2.1 mm. The experiment on the effect of the baffles was based on the MeOH/refined palm oil molar ratio of 6:1, reaction volume of 1000 mL, feed flow rate of 25 mL/min, NaOH loading of 1 wt% and at 62–63 °C. The result is shown in Fig. 1.

The presence of the baffles shows a significant effect on both steady-state FAME yield and energy consumption. The FAME yield increased from 72.5 to 80% after the reaction time of 90 min. Also, for the non-baffled reactor, at the same impeller speed, the temperature of the mixture rose very slowly as it took about 30 min to reach 62 °C. For the baffled reactor, only about 10 min was required. This finding confirms the hypothesis that the reactor with rapidly-spinning blades also requires baffles to help improve the heat and mass transfer, as well as flow pattern of liquid resulting in rapid temperature rise causing the endothermic reaction to move forward^34^. They work by disrupting the flow pattern and keeping the movement of the top to the bottom fluid to promote radially axial circulation of the fluid^37^. In addition, they prevent swirling and vortexing of liquid which results in a high shear rate^38^ and decrease the relative velocity between the impeller and liquid mixture resulting in enhancement of the degree of turbulence. This result agreed with the report of Metawea et al.^39^ who studied the effect of mesh baffles on the FAME yield using a batch agitated vessel and found that the installed baffles improved FAME yield significantly. In fact, from eye observation through the viewport, for the non-baffled case, most of the liquid mixture appears to be pushed to the side of the reactor and spin freely. As only a small portion of the mixture came into physical contact with the impeller, higher rotational energy was required to bring the overall mixture to the operating temperature compared to the baffled case.

#### Effects of reaction volume and reactant total flow rate on FAME yield

**Figure 2:**
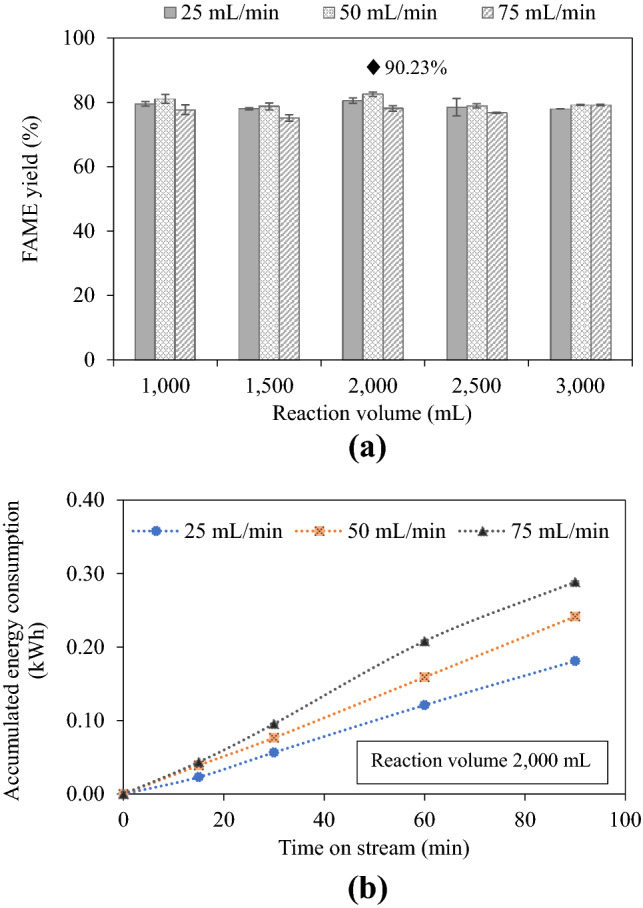
(**a**) Effects of reaction volume and total feed flow rate on steady-state FAME yield (refined palm oil, 1 wt% NaOH, 6:1 MeOH: oil molar ratio, 62–63 °C), ♦ = after washing and (**b**) accumulated energy consumption (2000 mL case).

Figure 2a shows the influence of reaction volume and total feed flow rate on FAME yield at a steady state. It is important to note that, by performing selected experiments for 210 min (see Supplementary Fig. S4), the FAME yield reached a steady state at around 60 min. The flow rate of 50 mL/min appears to provide the highest FAME yield for all mixture volumes studied. At this flow rate, FAME yield was increased when the reaction volume rose from 1000 to 2000 mL, and then dropped with a further increase in the reaction volume. Thus, the 2000 mL volume and the total feed flow rate of 50 mL/min are the optimal process parameters for the designed reactor giving 82.53% steady-state FAME yield and increasing to 90.23% after washing.

For a volume larger than 2000 mL, the concentration of microbubbles from cavitation decreases due to inactive zones at regions away from the blade tips^40^ as well as lower mass and heat transfer, leading to a declined FAME yield. The cavitational effect taking place inside the reactor is a crucial phenomenon for improving mixing efficiency. The rapidly spinning impeller causes a sufficient pressure reduction in the liquid generating numerous microbubbles. They flow through the tips and trailing edges of the spinning blades and they are then ripped into the surrounding fluid and eventually collapse and mix the reaction mixture well^41^. Due to the high rotational speed of the impeller, rapid liquid circulation enhances the mixing efficiency as well as increases the reaction kinetics. For the effect of total feed flow rate, the FAME yield for all reaction volumes increased with the increase in feed flow rate from 25 to 50 mL/min, but decreased with a higher flow rate of 75 mL/min. This result is in contrast to the study of Chen et al.^42^ and Santacesaria et al.^43^ as they reported that the increase in flow rate lowered the residence time leading to a lower biodiesel yield. However, the present work controlled the temperature inside the chamber by adjusting the speed of the impeller. When the feed flow rate increased, the mixture temperature decreased, and the impeller must turn faster to maintain the same temperature. It might be concluded that the agitation speed has a greater effect on the biodiesel yield.

For the reaction volume of 2000 mL, the three total feed flow rates of 25, 50 and 75 mL/min correspond to the residence time of 80, 40 and 26.67 min, respectively. As the optimum flow rate was 50 mL/min, the optimum residence time for the studied reactor is 40 min. Darnoko and Cheryan^24^ also found an optimum residence time of 60 min to achieve the highest concentration of esters of 85.6% based on a CSTR reactor, with shorter or longer residence time resulting in low yield. The effect of residence time in the present work can be discussed as follows. The longer residence time provides the higher FAME yield, according to the literature that methyl esters content and conversion of oil from transesterification increase with increasing time^24,44^. In addition, the residence time for continuous transesterification could exhibit wide ranges from seconds to minutes depending on reactor types and flow rate. Long residence time could enhance the hydrolysis of esters causing ester loss and lowering FAME yield^45^. Too much residence time could also lead to the reversible reaction and methanol evaporation, reducing the overall yields^10,12,46^. The residence time of the present work was still shorter than that of the batch stirred reactor of 1 h and the membrane reactor of 1–3 h^47^, indicating higher production efficiency. For the accumulated energy consumption shown in Fig. 2b, a higher flow rate requires more energy to heat an additional quantity of reactants entering the reactor. Energy consumption will have an effect on yield efficiency.

An appropriate design of a cylindrical chemical reactor with respect to height and diameter helps increase heat transfer, liquid mixing as well as productivity. Conventionally, the optimum ratio of the height (*H*) to diameter (*D*) of a cylindrical chemical reactor is unity. The height of the solution mixture in the reactor for the 2000 mL case is about 11.5 cm (*H*). With the reactor diameter of 15 cm (*D*), the *H*/*D* ratio is approximately 0.8. The *H*/*D* ratio of unity would correspond to the mixture volume of roughly 2120 mL. The slight inconsistency between the optimal *H*/*D* ratio of the designed reactor and the theory may indicate that a cylindrical reactor with very high impeller speed may not follow the theory applied for conventional cylindrical chemical reactors with low impeller speed.

#### Effect of reactant molar ratio on FAME yield

**Figure 3:**
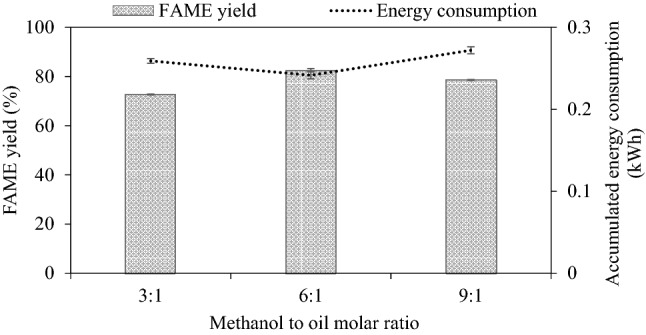
Effect of reactant molar ratio on steady-state FAME yield (refined palm oil, 2000 mL, 50 mL/min, 1 wt% NaOH, 62–63 °C).

Stoichiometrically for transesterification, 1 mol of triglyceride requires 3 mol of alcohol to generate 3 mol of FAME and 1 mol of glycerol. However, a high amount of alcohol is preferred to push the reaction forward for a very fast reaction rate and enhance the oil conversion. Also, at a lower methanol ratio, the high-viscosity oil needs a higher amount of methanol to be soluble in methanol^48^. In this work, the methanol to refined palm oil molar ratios of 3:1, 6:1 and 9:1 with 1 wt% of NaOH at reaction volume of 2000 mL, feed flow rate of 50 mL/min, and at 62–63 °C were investigated and the results are depicted in Fig. 3. The steady-state FAME yield is low for the stoichiometric ratio of 3:1 due to the lowering in miscibility and contacting between molecules of methanol and oil^48^. When increasing the molar ratio to 6:1, the FAME yield increased from 72.69 to 82.53%. Beyond the 6:1 molar ratio, the excess amount of methanol slightly decreased the steady-state FAME yield to 78.58%. The cause of the decline in FAME yield could be due to the enhancement of solubility of methanol, FAME and glycerol. A higher amount of methanol also lowered the concentration of oil and decreased the FAME yield. Besides, there was a report indicating that a ratio of methanol to oil higher than 6:1 negatively affected the FAME yield due to the formation of glycerol emulsion with produced FAME leading to a smaller yield^39^. This result corresponded to the report that biodiesel yield can decrease due to excess methanol^39,45^. In addition, the use of excess methanol causes a high cost of methanol recovery and separation. Hence, the methanol to oil molar ratio of 6:1 was an optimum value for further studies. This ratio is also beneficial for yield efficiency as the accumulated energy consumption is minimal at this ratio.

#### Effect of catalyst concentration on FAME yield

**Figure 4:**
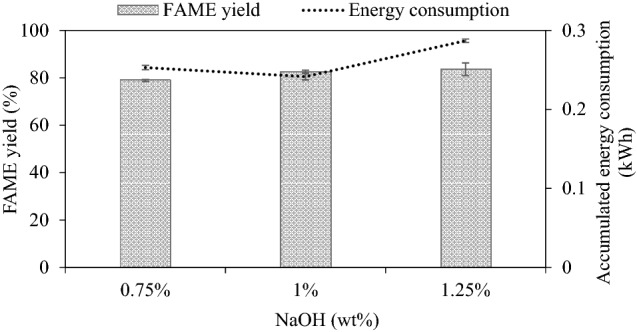
Effect of catalyst loading on steady-state FAME yield (refined palm oil, 2000 mL, 50 mL/min, 62–63 °C).

Based on the results in Fig. 4, the steady-state FAME yield increased with more catalyst loading from 0.75 to 1.25 wt%, reaching the highest value of 83.61% for 1.25 wt% NaOH. However, the yield increase of only 1.08% by increasing the catalyst concentration from 1 to 1.25 wt% does not justify using the high catalyst loading. At the low catalyst content of 0.75 wt.%, a low FAME yield was observed. A small amount of catalyst means a small number of active sites present to react with oil, resulting in a low FAME yield^40^. Also at 1.25 wt%, emulsion formation was observed, and this phenomenon leads to difficulty in separation^15^ including increased production and purification costs. The higher mixture viscosity for the 1.25 wt% case is evident from the substantial increase in the accumulated energy consumption. Therefore, the 1 wt% catalyst concentration is appropriate for the studied reactor design. It is also beneficial for yield efficiency as the accumulated energy consumption is minimal at this catalyst loading.

#### Effect of reaction temperature on FAME yield

**Figure 5:**
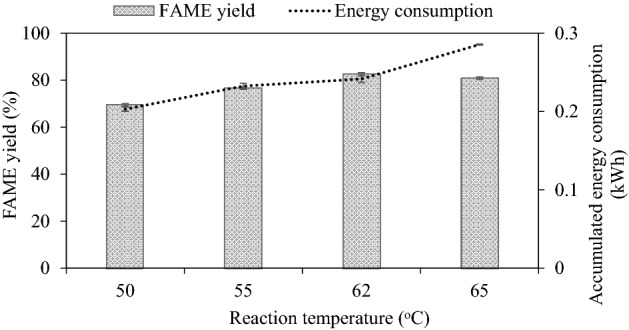
Effect of reaction temperature on steady-state FAME yield (refined palm oil, 2000 mL, 50 mL/min, 1 wt% NaOH).

Reaction temperature affects kinetics and equilibrium. Higher temperatures can increase heat transfer and reduce the viscosity of the oil. The results shown in Fig. 5 indicate that as the reaction temperature increased from 50 to 62 °C, the FAME yield at steady state was enhanced and that at 65 °C, the yield dropped. The maximum steady-state FAME yield of 82.53% was obtained at 62 °C. At low reaction temperature, the rate of reaction was slow^49^ resulting in a low FAME yield. For excess temperature (65 °C) which is higher than the boiling point of methanol (64.7 °C), it promotes methanol being in a vapor phase while the oil is still in a liquid phase affecting poor contact between the reactants^15^ and changes the optimal molar ratio of methanol to oil. High reaction temperature also affects energy consumption negatively.

#### Effect of reactant discharge position on FAME yield

**Figure 6:**
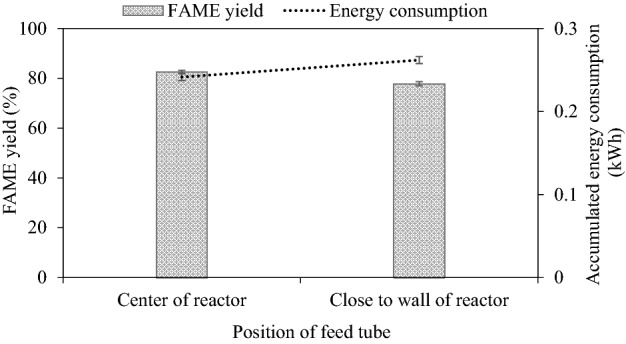
Effect of reactant discharge position on steady-state FAME yield (refined palm oil, 2000 mL, 50 mL/min, 1 wt% NaOH, 62–63 °C).

The position of the reactant discharge is important for liquid-phase reactions. The influence of reactant discharge position is shown in Fig. 6. The centered position showed better steady-state FAME yield than the against-wall position (82.5% vs 77.8%). At the center or near the tip of the impeller positions which delivered the highest turbulence^50^, the new reactant experienced effective mixing to overcome the less solubility of oil and methanol which gave the best result of FAME yield. In general, the new feed mixed the fastest with the existing fluid at a strong turbulence region or where the shortest local mixing time constant existed^50^. When the discharge position is at the reactor wall, besides not mixing well in every direction, a certain amount of the new reactants would quickly flow out of the discharge port without undergoing transesterification, a phenomenon known as short-circuiting. It is also important to note that the energy consumption was slightly higher for the off-centered case too.

### FAME production based on various types of edible vegetable oil

**Figure 7:**
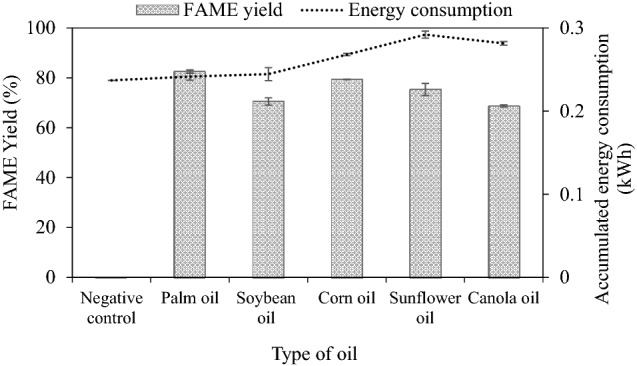
Effect of various types of vegetable oils on steady-state FAME yield (2000 mL, 50 mL/min, 1 wt% NaOH, 62–63 °C).

Figure 7 illustrates the results of transesterification of each oil type. It is seen that palm oil as a feedstock showed the highest steady-state yield of 82.53% (increasing to 90.23% after washing). Corn oil and sunflower oil gave slightly lower FAME yield at steady state, while soybean oil and canola oil gave the lowest methyl esters content. Literature review reveals that fatty acid components of each feedstock do not change notably throughout transesterification while physical and chemical properties of FAME depend on chain length and number of double bonds of feedstocks^6^. Although soybean, sunflower and corn oils contain a similar degree of unsaturated carbon bonds in contrast to palm oil, the steady-state FAME yields are different. Thus, FAME yield does not depend on the degree of saturation of the oil. In fact, the molecular weight of oils directly relates to carbon chain length^51^. When considering in terms of molecular weight of each feedstock (see Supplementary Table S1), palm oil which comes with the lowest molecular weight gave the highest FAME yield while corn oil consisting of the second lowest molecular weight provided the second highest FAME yield. Sunflower oil and canola oil have similar molecular weights, but the FAME yields are contrasting. Soybean oil comes with the highest molecular weight and gave the second lowest FAME yield. This result might be explained that the low molecular weight oil has less powerful steric hindrance and, in some cases, increased reactivity^52^ affecting the high conversion of oil. Moreover, the kinematic viscosity of each feedstock listed in Supplementary Table S1 might affect FAME yield. Palm oil showed a higher kinematic viscosity and a lower molecular weight corresponding to a lesser hindrance effect giving the highest FAME yield, while canola oil and sunflower oil had lower kinematic viscosity and higher molecular weight presenting lower FAME yield^40^. It can be concluded that, based on this study, FAME yield depends on the molecular weight (chain length) of feedstocks and the kinematic viscosity.

The type of feedstock used should depend on the availability in each region. Because the properties of biodiesel depend on the feedstock's fatty acid characteristics^6^, biodiesel from different feedstock types is suitable for use in certain climates. Palm oil is reported to contain high monounsaturated and saturated fatty acids which present superior oxidation stability but rather having poor cold flow properties^53^. Biodiesel derived from palm oil can be used in tropical regions without any problems except in cold weather due to fuel crystallization and precipitation leading to clogging in fuel lines, filters, and injectors of the engine^6,53^. Whereas biodiesel based on high polyunsaturated fatty acid content such as soybean, sunflower, and corn oils shows good cold flow characteristics suitable for cold-weather countries^53^. Therefore, the choice of feedstock in biodiesel production for each country is important for it affects the properties of biodiesel.

### FAME production based on WCO

**Figure 8:**
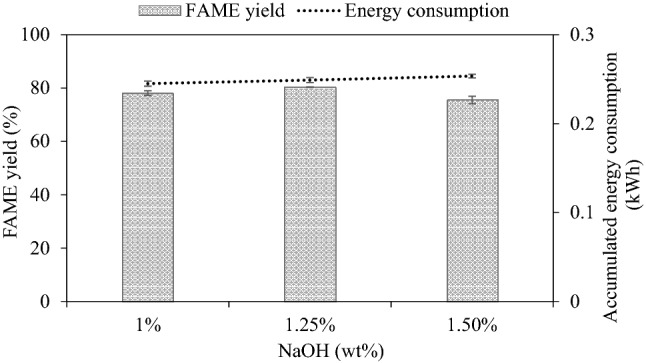
Effect of catalyst loading on WCO-derived FAME yield at steady state (2000 mL, 50 mL/min, 1 wt% NaOH, 62–63 °C).

As the price of edible oils contributes approximately 80% of biodiesel price^25^, the biodiesel production cost can be lowered by using WCO instead of refined vegetable oils. The effect of NaOH concentration on steady-state FAME yield is illustrated in Fig. 8. The FAME yield at steady state increased with increasing NaOH content from 1 to 1.25% decreased with a higher catalyst loading though. The highest steady-state FAME yield was 80.35% (increasing to 87.76% after washing). As expected, low catalyst concentration resulted in low FAME yield as there was an insufficient number of active sites to react. On the other hand, too much catalyst (1.5%) increased the saponification with triglycerides resulting in low FAME yield^25^ and biodiesel will turn solid, which is unsuitable for actual use. This result corresponds to the study of Maddikeri et al.^19^ who studied the intensification reaction of WCO through hydrodynamic cavitation at a catalyst loading of 0.75–1.25%. The result showed that too much catalyst concentration did not offer a remarkable increase in FAME yield.

### Influence of reactor insulation on steady-state FAME yield

**Figure 9:**
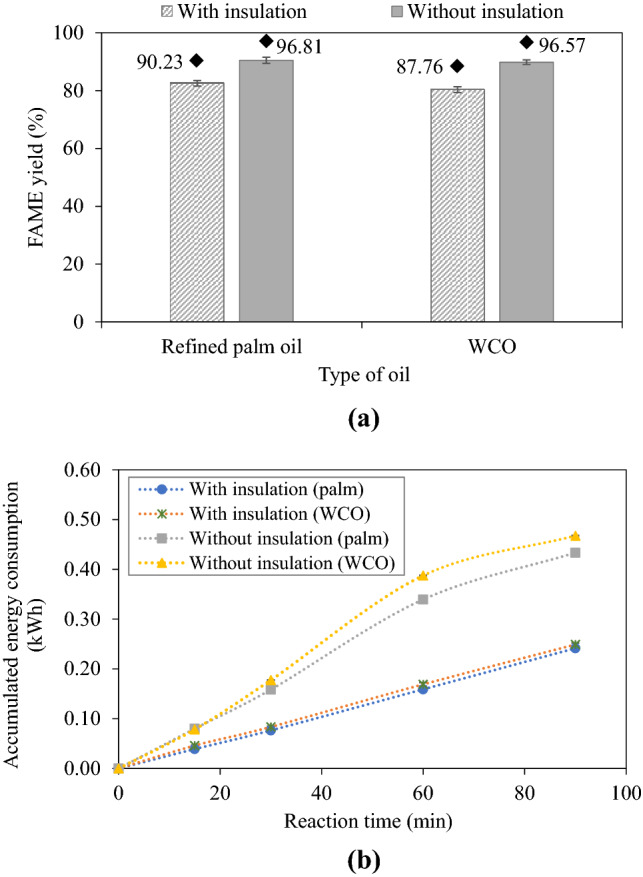
(**a**) Effect of insulation on steady-state FAME yield and (**b**) accumulated energy consumption (2000 mL, 50 mL/min, 1 wt% NaOH, 62–63 °C), ♦ = after washing.

The results of the effect of reactor insulation on steady-state FAME yield are shown in Fig. 9a. The steady-state FAME yields of 90.5% from refined palm oil and 89.85% from WCO were higher than those of the insulated reactor significantly. After washing, the yields further increased to 96.81 and 96.57% from refined palm oil and WCO, respectively. For the non-insulated reactor, as it continuously loses heat to the environment, in order to keep the bulk temperature between 62 and 63 °C, the speed of the impeller must be higher than that of the insulated reactor resulting in a higher FAME yield. This effect is evident from the higher accumulated energy consumption shown in Fig. 9b. The higher viscosity of WCO than refined palm oil contributed to the higher energy consumption as well. By maintaining the same reaction temperature for both insulated and non-insulated reactor cases, the result signifies the effect of the impeller speed. The higher impeller speed also increases the number of numerous microbubbles from cavitation occurrence which improves mass and heat transfer, as well as reactant homogenization. Besides, when the impeller speed is high, oil dispersion in the methanol phase containing the catalyst is enhanced^40^, resulting in a higher FAME yield. Nevertheless, more heat accumulation could be generated with increasing impeller speed^40^. This also suggests that even higher steady-state FAME yield could be achieved by active cooling of the reactor, e.g., water cooling, so that higher impeller speed can be applied while maintaining the same reaction temperature of 62–63 °C. The high impeller speed of about 17,900 rpm together with the calculated Reynolds number^54^ of 19,400 indicated that it was a fully turbulent operation. Hence, this can be implied that the mixture between oil and methanol is well-mixed throughout the experiment^55^. The result of insulation associated with impeller speed conforms to literature reports. For example, Metawea et al.^39^ and Peiter et al.^56^ obtained the same results and described that stirring speed notably affected the transesterification reaction. Nonetheless, as transesterification is an endothermic reaction in nature^57^, operating with insulation could hinder heat dissipation from the reaction mixture resulting to shift the reaction forward.

This finding suggests that the FAME yield of waste cooking oil as feedstock is as good as the yield of FAME based on palm oil. Therefore, the use of waste cooking oil is another promising sustainable alternative for continuous biodiesel production. In addition, in small communities, the waste oil from cooking can be collected and used to produce biodiesel. It is one way to reduce waste to the environment, as well as to increase the added value of waste oil.

### The first-order kinetics for transesterification of refined palm oil and WCO

The reaction kinetics was evaluated at 50, 55, 62 and 65 °C. The kinetics was studied with the residence time between 0 and 40 min. By fitting the experimental data using Eq. (2), a good linear relationship between $\frac{{X_{A} }}{{\left( {1 - X_{A} } \right)}}$ and $t$ indicates that the first-order kinetics is suitable for transesterification of both refined palm oil and WCO.

The kinetic rate constant (*k*) and R^2^ obtained from transesterification are listed in Supplementary Table S3. The highest rate constant of 0.1204 min^−1^ with R^2^ = 0.9801 of palm oil transesterification was found at 62 °C, confirming that the reaction rate at 62 °C is fastest and it follows the first-order kinetics which is in good agreement with the literature^33^. Furthermore, *k* and R^2^ achieved from WCO transesterification were 0.1155 min^−1^ and 0.9934, respectively. It was found that the kinetic rate constant of transesterification of refined palm oil was slightly higher than that of WCO. This might be due to the presence of some impurity compounds in WCO that could obstruct the transesterification rate^58^. Darnoko and Cheryan^59^ studied the kinetics of palm oil transesterification in a batch reactor and found the reaction rate constant at 60 °C of 0.141 min^−1^ to obtain a biodiesel yield of 90%. Pauline and the group^60^ reported the reaction rate constant obtained at 60 °C of waste cooking oil transesterification as 0.032 min^−1^ with the maximum biodiesel yield of 90%.

### Activation energy

The activation energy of the studied transesterification derived from refined palm oil obtained from plotting of ln(*k*) and 1/*T* as shown in Supplementary Fig. S5a was 29.38 kJ/mol. The Arrhenius equation based on refined palm oil as a feedstock for the present study can be expressed as Eq. (4):

4 $$\ln \left( k \right) = ~ - 3533.3/T + 8.3$$

The activation energy of WCO transesterification was found to be 35.31 kJ/mol as shown in Supplementary Fig. S5b. The Arrhenius equation can be expressed as Eq. (5):

5 $$\ln \left( k \right) = ~ - 4246.8/T + 10.4$$

The activation energy refers to the minimum energy required for a reaction to occur. In general, the activation energy for homogeneous alkali-catalyzed transesterification of palm oil should be in the range of 27.3–61.5 kJ/mol^59^. In this work, the calculated activation energies for both palm oil and WCO are within this range and are comparable to the work of Issariyakul et al.^61^ who reported the activation energy of 30.2 kJ/mol for palm oil transesterification with a homogeneous catalyst, as well as the work of Pauline et al.^60^ who presented the activation energy of 27.24 kJ/mol for waste cooking oil transesterification.

### FAME properties

Properties of FAMEs produced from refined palm oil and WCO at the optimum conditions are listed in Table 1. All of the analyzed properties follow the EN 14214 and ASTM D6751 standards.

**Table 1:** Properties of produced FAMEs.

Property	Synthesized FAME from refined palm oil	Synthesized FAME from WCO	EN 14214	ASTM D6751
Density (g/cm^3^)	0.87	0.88	0.85–0.90	0.86–0.90
Kinematic viscosity (40 °C, mm^2^/s)	4.19	4.20	3.5–5.0	1.9–6.0
Cloud point (°C)	12	11	–	−3 to 12
Acid value (mg of KOH/g of oil)	0.39	0.5	≤ 0.5	≤ 0.5
FAME yield (%) (without insulation)	96.81	96.57	≥ 96.5	–

### Yield efficiency

The yield efficiency of FAME derived from refined palm oil and WCO of selected pieces of literature is reported in Supplementary Table S4 which summarizes the yield efficiency of each technology reactor. The yield efficiency of biodiesel derived from refined palm oil was 21.1 × 10^–4^ g/J while the yield efficiency of biodiesel derived from WCO was 19.4 × 10^–4^ g/J. When comparing to values obtained by other researchers using other intensified technologies, the yield efficiency of the present work is higher.

Appamana et al.^22^ performed continuous biodiesel production based on refined palm oil using an intensified spinning disc reactor (SDR) and evaluated the biodiesel yield and yield efficiency, which were found to be 97% and 13.7 × 10^–4^ g/J, respectively, with the residence time of 3 s. From the energy efficiency point of view, it was lower than the value of the present study. A hydrodynamic cavitation reactor was used to perform transesterification of rubber seed oil and WCO by Bokhari et al.^62^, Chuah et al.^41^ and Maddikeri et al.^19^ in a batch system. They reported synthesized FAME yield of 88, 98 and 89.24%, respectively, while the yield efficiency values were 12.5, 12.5 and 12.2 × 10^–4^ g/J, respectively. Maddikeri et al.^63^ studied intensification of interesterification from WCO with methyl acetate through a batch ultrasonic system. The system had an irradiation frequency of 22 kHz and rated power of 750 W. The result revealed the maximum yield (90%) of biodiesel obtained from a very high molar ratio of oil: methyl acetate of 1:12, catalyst concentration of 1% and temperature of 40 °C for 30 min with the yield efficiency of 2.1 × 10^–4^ g/J. It was suggested that the yield efficiency of the proposed food blender was 1.5, 1.7 and 10 times greater than that of the spinning disc reactor, hydrodynamic cavitation reactor and ultrasonic reactor, respectively. Compared to the above-mentioned pieces of literature, it is interesting to note that the continuous system requires less energy consumption than a batch system. The calculated yield efficiency from the proposed food blender shows an advantage in terms of the lower energy requirements and feasibility in the economic point of view to be used in small communities.

### Preliminary design of biodiesel production system for small communities

The present work successfully demonstrates the continuous biodiesel production through the upgraded kitchen blender with a production rate of 3 L an hour (with no purification process). The biodiesels produced from both refined palm oil and WCO meet the world’s standards and can be blended with petro-diesel for use in diesel engines. The proposed production system for small communities can be very simple. Instead of large tanks and equipment pieces for a conventional batch process requiring a substantial footprint, several of such continuous reactors can be operated in parallel. Each unit, a reactor connected to a kitchen blender, can be placed on a shelf to take advantage of the small height of each unit. Thus, the total area required for transesterification would be very small. For instance, if 30 units were to be operated in parallel, the total area required would be only that of a medium-sized shelf with 3 stories (assuming 10 units on each story) as illustrated in Supplementary Fig. S6. The production capacity would be as high as 90 L/h, which would be more than sufficient for a small community. Only two pumps would be required, one for oil and the other for MeOH + NaOH, to provide feeds to all units through split valves. The system could easily be expanded by installing more units to increase the production capacity for medium-sized communities or even small enterprises. Another good aspect of running such units in parallel is that if one unit malfunctioned, it could be easily and quickly replaced without affecting the entire production process. In terms of yield efficiency, the present research showed better results, 21.1 × 10^–4^ g/J for refined palm oil and 19.4 × 10^–4^ g/J for WCO, than that of commercial biodiesel plants in Thailand of 5.6 × 10^–4^ g/J^64^ and produced biodiesel in Iran of 0.4 × 10^–4^ g/J^65^, signifying that the proposed kitchen blender can be more energy efficient than conventional productions. In terms of capital investment, each unit would cost no more than USD 50. The two fluid pumps with low pumping heads would cost no more than USD 200. The whole production system would cost less than USD 2000, thus, the capital investment is low. It may also be possible to gravity drain the two fluids from their tanks, eliminating the use of the two pumps, but the flow rates would not be constant unless the two fluid levels were kept relatively constant during the production. This novel yet very simple, low-cost biodiesel production technology could replace large production plants.

## Conclusions

The present research successfully demonstrates the operation of the modified high-power food blender for continuous transesterification of refined vegetable oils and waste cooking oil for small community uses. The 1200 W household food blender was equipped with a stainless steel chemical reactor (15 cm in diameter and 19.8 cm in height). Two peristaltic pumps were continuously employed to inject oil and methanol into the center of the reactor. The temperature of the liquid mixture was regulated by the impeller speed controlled by a variac. The product was discharged from the discharge port at the desired level. Results indicated that the optimal conditions were: 2000 mL reaction volume, 50 mL/min total flow rate, 1% and 1.25% catalyst concentration for refined palm oil and WCO, respectively, 6:1 methanol-to-oil molar ratio and 62–63 °C. The highest obtained steady-state FAME yields were 96.81% (for palm oil) and 96.57% (for WCO). All the properties of produced FAMEs meet the EN 14214 and ASTM D6751 standards. The activation energy of the studied transesterification of refined palm oil and WCO was 29.38 and 35.31 kJ/mol, respectively. This simple continuous food blender can be operated in a parallel configuration to produce more biodiesel for small communities with high yield efficiency and low capital investment.

## Supplementary Information

Supplementary Information.
