# Supplementary material for: Low-cost alternative biodiesel production apparatus based on household food blender for continuous biodiesel production for small communities
Source: Sci Rep. 2021 Jul 5;11:13827. doi: 10.1038/s41598-021-93225-5 (PMC8257631; doi:10.1038/s41598-021-93225-5)
Supplement: Supplementary file 1 — Supplementary Information. [file 41598_2021_93225_MOESM1_ESM.docx]

**Low-Cost Alternative Biodiesel Production Apparatus Based on Household Food Blender for Continuous Biodiesel Production for Small Communities**

Wijittra Wongjaikham^a^, Doonyapong Wongsawaeng^a,*^, Vareeporn Ratnitsai^b^,

Manita Kamjam^a^, Kanokwan Ngaosuwan^c^, Worapon Kiatkittipong^d^, Peter Hosemann^e^, Suttichai Assabumrungrat^f,g^

*^a^ Research Unit on Plasma Technology for High-Performance Materials Development, Department of Nuclear Engineering, Faculty of Engineering, Chulalongkorn University, Bangkok 10330, Thailand*

*^b^ Department of Science and Mathematics, Faculty of Science and Technology, Rajamangala University of Technology Tawan-ok, Chonburi 20110, Thailand*

*^c^ Division of Chemical Engineering, Faculty of Engineering, Rajamangala University of Technology Krungthep, Bangkok 10120, Thailand^b^ Division of Chemical Engineering, Faculty of Engineering, Rajamangala University of Technology Krungthep, Bangkok 10120, Thailand*

*^d^ Department of Chemical Engineering, Faculty of Engineering and Industrial Technology, Silpakorn University, Nakhon Pathom 73000, Thailand*

*^e^ Department of Nuclear Engineering, Faculty of Engineering, University of California at Berkeley, 94720, U.S.A.*

*^f^ Center of Excellence in Catalysis and Catalytic Reaction Engineering, Department of Chemical Engineering, Faculty of Engineering, Chulalongkorn University, Bangkok 10330, Thailand*

*^g^ Bio-Circular-Green-economy Technology & Engineering Center, BCGeTEC, Faculty of Engineering, Chulalongkorn University, Bangkok, Thailand 10330*

* Corresponding author. Doonyapong.W@chula.ac.th., Department of Nuclear Engineering, Faculty of Engineering, Chulalongkorn University, 254 Phayathai Road, Pathumwan, Bangkok 10330, Thailand.

This supplementary information includes:

Supplementary Text

Figure S1-S6 with caption

Table S1-S4 with caption

References

**Characteristic of impeller**


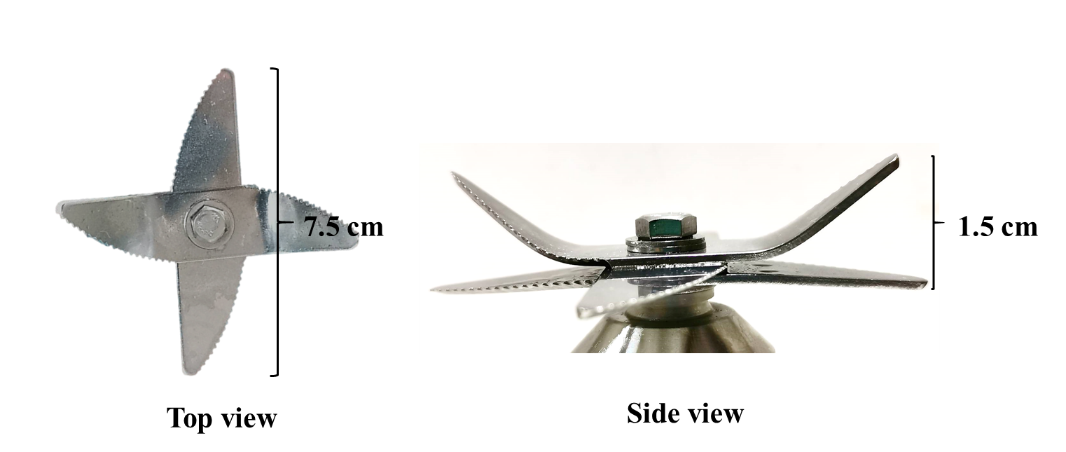


**Fig. S1.** Characteristic of impeller

The configuration of the impeller is shown in Fig. S1. The impeller is a wide-blade impeller type (propeller) consisting of 3 sets of 2 blades each (total 6 blades). One side of each blade on the direction of the rotation exhibits a sharp, serrated teeth-like feature while the opposite side is smooth. The impeller is obviously designed to cut materials; however, as it was proven by Wongsawaeng et al. [1], the impeller is very effective in mixing vegetable oils and methanol as well. The ratio of the impeller diameter (D) to the tank diameter (T) should be in the range 0.4 - 0.6. As the impeller diameter is 7.5 cm and as the reactor diameter is 15 cm, the D/T ratio of the present reactor is 0.5, which is proper.

**Experimental set up**


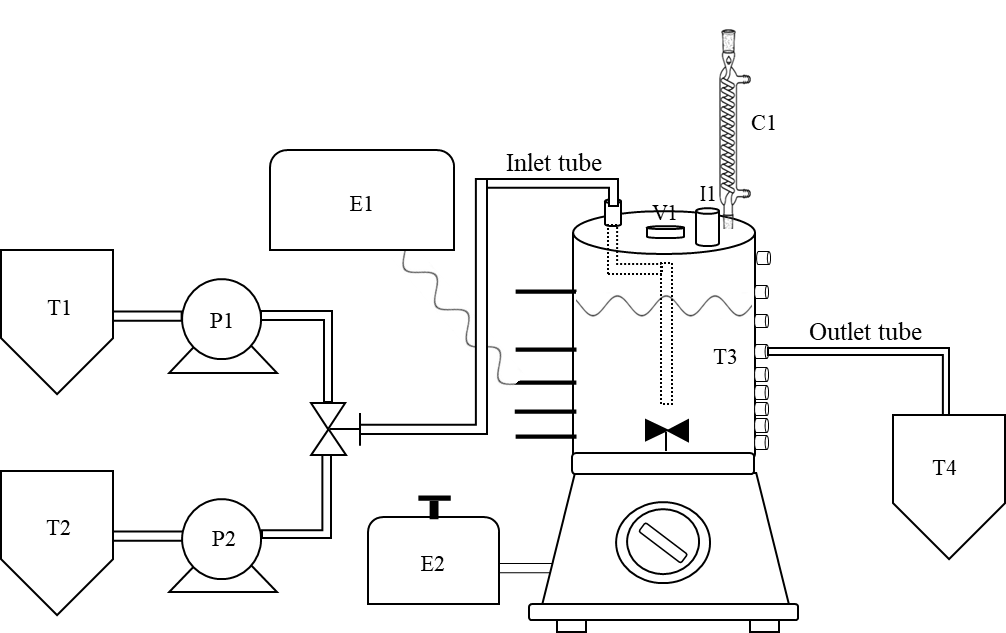


**Fig. S2.** Schematic diagram of continuous FAME production (T1: oil tank, T2: methanol + NaOH tank, T3: chemical reactor, T4: discharge tank, P1: peristaltic pump for oil tank, P2: peristaltic pump for methanol tank, E1: thermocouple reader connected to a thermocouple, E2: variac, V1: viewport, I1: inlet port, C1: condenser)

Fig. S2 shows a schematic diagram of the setup for continuous transesterification aiming for household utilization, so it was kept as simple and as convenient as possible. The oil and the mixture of methanol with dissolved catalyst were co-fed into an inlet port on the lid of the reactor by peristaltic pumps. The fluid temperature in the reactor was measured by thermocouples installed on the side of the reactor. The voltage supplied to the food blender was regulated by a variac to control the impeller speed and the reaction temperature. A plug-in power meter was used to measure the instantaneous and accumulation of energy consumption to allow yield efficiency calculation. The liquid mixture naturally discharges from the reactor by overflowing through an open outlet port at the elevation from the bottom of the reactor corresponding to studied volume.

**
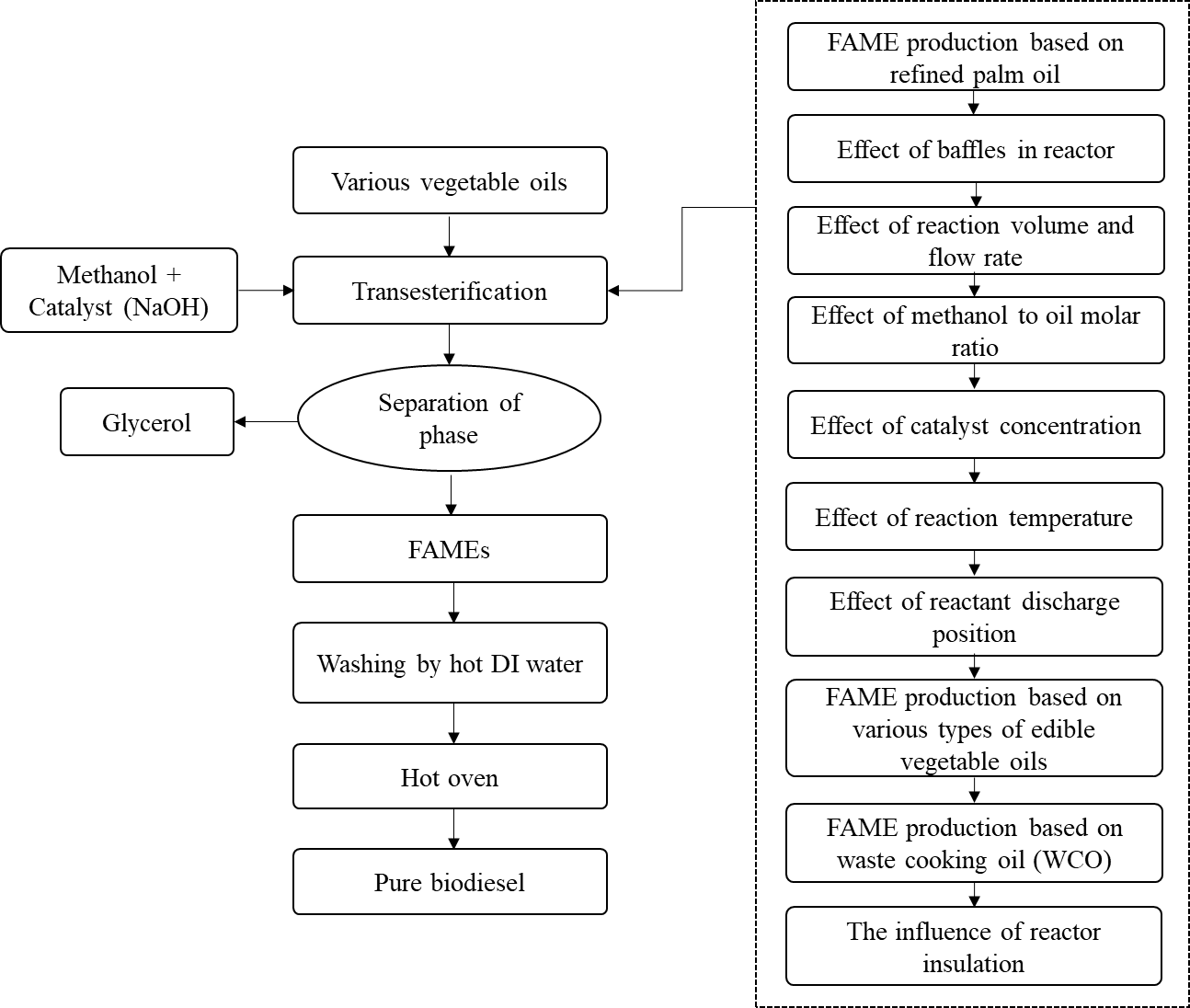
**

**Fig. S3.** Schematic of overall biodiesel production process

**Determination of fuel properties**

*Density [2]*

The density of the produced FAME was measured in the laboratory corresponding to the ISO 4787 standard at 30^o^C using a pycnometer. The tests were repeated two times and the average value was reported.

*Kinematic viscosity [2]*

The kinematic viscosity was determined at 40^o^C through the ASTM D445 standard by means of a viscometer. FAME was heated using a heating tape instead of a water bath, and a falling time of the moving liquid between the two horizontal lines marked on the viscometer tube was measured. The experiments were performed three times for each sample.

*Cloud point*

The cloud point was measured following the ASTM D 2500 [3] standards using Walter Herzog GmbH, Germany equipment.

*Acid value*

The acid value indicates the presence of acidity in FAME determined by the AOCS method by titration. A quantity of 2 g of biodiesel sample was dissolved in 30 mL isopropyl alcohol. The mixture was boiled for 2 min and titrated with a 0.1 N KOH solution with phenolphthalein as a color indicator [4]. Acid value can be evaluated from Eq. (1).

Acid value = $\frac{\left( S-B \right) \times N \times56.1}{Weight of sample (g)}$ (1)

where S is the titrant (KOH) volume used for the sample (mL), B is the titrant volume used for the blank (mL), and N stands for normality of the KOH standard.

**Results and discussion**

(a)

(b)

**Fig. S4** FAME yield over reaction time (a) feed flow rate of 25 mL/min (b) feed flow rate of 50 mL/min

(a)

(b)

**Fig. S5.** Arrhenius plot of studied transesteriﬁcation of (a) refined palm oil and (b) WCO


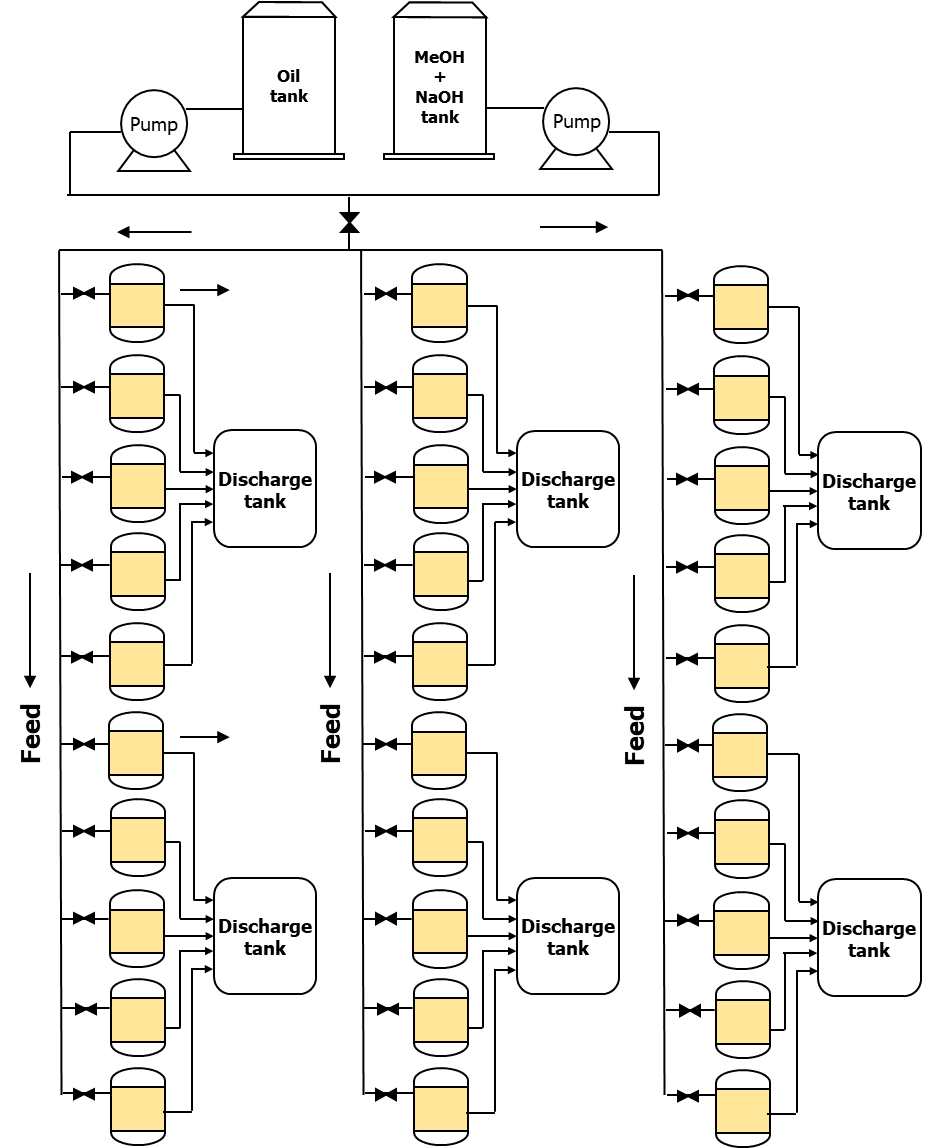


**Fig. S6.** Preliminary model of biodiesel production system for small communities

**Table S1** Properties of fresh refined vegetable oils and WCO

| Type of oil | Density (g/cm^3^) | Molecular weight (g/mol) | FFA content (%) | Acid value  (mg of KOH/g of oil) | Kinematic viscosity  (40^o^C, mm^2^/s) |
| --- | --- | --- | --- | --- | --- |
| Palm oil | 0.89 | 847 | 0.10 | 0.20 | 40.21 |
| Soybean oil | 0.91 | 920 | 0.11 | 0.21 | 32.54 |
| Corn oil | 0.91 | 865.4 | 0.16 | 0.32 | 29.08 |
| Sunflower oil | 0.92 | 876.2 | 0.12 | 0.24 | 32.15 |
| Canola oil | 0.92 | 876.6 | 0.13 | 0.28 | 32.18 |
| WCO | 0.90 | 845 | 1.21 | 2.60 | 41.26 |

All Data based on experiments

**Table S2** A salient design details of the equipment

| Equipment | Specification |
| --- | --- |
| Household food blender power | 1,200 W |
| Cylindrical-shaped chemical reactor material | 304 stainless steel |
| Maximum capacity of chemical reactor | 3, 500 mL |
| Chemical reactor dimension  (D × H) | 15 × 19.8 cm |
| Impeller dimension (L× H) | 7.5 × 1.5 cm |
| Baffle dimension (L× W) | 19.8 × 1.3 cm |
| Reactant injection port diameter | 0.3 cm |
| Discharge port diameter | 0.5 cm |

**Table S3** First-order rate constants for transesteriﬁcation of refined palm oil and WCO at different temperatures (2,000 mL, residence time 0 - 40 min, 1 wt% NaOH for refined palm oil and 1.5 wt% NaOH for WCO)

| T (^o^C) | Refined palm oil | | WCO | |
| --- | --- | --- | --- | --- |
|  | *k* (min^-1^) | R^2^ | *k* (min^-1^) | R^2^ |
| 50 | 0.0719 | 0.9637 | 0.0607 | 0.9824 |
| 55 | 0.0845 | 0.9741 | 0.084 | 0.9631 |
| 62 | 0.1204 | 0.9801 | 0.1155 | 0.9934 |
| 65 | 0.1065 | 0.9971 | 0.1036 | 0.983 |

**Table S4** Yield efficiency of FAME from intensification technologies

| **Ref.** | **Yield efficiency**  **(× 10^-4^ g/J)** | **Type of reactor** | **Mode of operation** |
| --- | --- | --- | --- |
| Present work  (refined palm oil) | 21.1 | High-power food blender | Continuous |
| Present work  (WCO) | 19.4 | High-power food blender | Continuous |
| Wongsawaeng et al. [1] | 45.2 | Household blender | Batch |
| Appamana et al. [5] | 13.7 | Spinning disc | Continuous |
| Bokhari et al. [6] | 12.5 | Hydrodynamic cavitation | Batch |
| Chuah et al. [7] | 12.5 | Hydrodynamic cavitation | Batch |
| Maddikeri et al. [8] | 12.2 | Hydrodynamic cavitation | Batch |
| Maddikeri et al. [9] | 2.1 | Ultrasound | Batch |

**References**

1. Wongsawaeng, D., et al., Simple and effective technology for sustainable biodiesel production using high-power household fruit blender. *Journal of Cleaner Production*. **237**, 117842 (2019).

2. Gülüm, M. and A. Bilgin, Measurements and empirical correlations in predicting biodiesel-diesel blends’ viscosity and density. *Fuel*. **199**, 567-577 (2017).

3. Tesfaye, M. and V. Katiyar, Microwave assisted synthesis of biodiesel from soybean oil: Effect of poly (lactic acid)-oligomer on cold flow properties, IC engine performance and emission characteristics. *Fuel*. **170**, 107-114 (2016).

4. Chapter 9 - Analytical Methods, in *Fats and Oils Handbook* (ed. Bockisch, M.) 803-808 (AOCS Press.1998).

5. Appamana, W., et al., Intensification of Continuous Biodiesel Production Using a Spinning Disc Reactor. *Journal of chemical engineering of Japan*. **52**, 545-553 (2019).

6. Bokhari, A., et al., Pilot scale intensification of rubber seed (Hevea brasiliensis) oil via chemical interesterification using hydrodynamic cavitation technology. *Bioresource Technology*. **242**, 272-282 (2017).

7. Chuah, L.F., et al., Intensification of biodiesel synthesis from waste cooking oil (Palm Olein) in a Hydrodynamic Cavitation Reactor: Effect of operating parameters on methyl ester conversion. *Chemical Engineering and Processing: Process Intensification*. **95**, 235-240 (2015).

8. Maddikeri, G.L., P.R. Gogate, and A.B. Pandit, Intensified synthesis of biodiesel using hydrodynamic cavitation reactors based on the interesterification of waste cooking oil. *Fuel*. **137**, 285–292 (2014).

9. Maddikeri, G.L., A.B. Pandit, and P.R. Gogate, Ultrasound assisted interesterification of waste cooking oil and methyl acetate for biodiesel and triacetin production. *Fuel Processing Technology*. **116**, 241-249 (2013).
